# Resultados Precoces do Procedimento de Norwood em um Centro de Referência no Brasil

**DOI:** 10.36660/abc.20201226

**Published:** 2022-06-06

**Authors:** Rodrigo Freire Bezerra, Juliana Torres Pacheco, Sônia Meiken Franchi, Rosangela Belbuche Fittaroni, José Francisco Baumgratz, Rodrigo Moreira Castro, Luciana da Fonseca da Silva, José Pedro da Silva

**Affiliations:** 1 Hospital Beneficência Portuguesa de São Paulo São Paulo SP Brasil Hospital Beneficência Portuguesa de São Paulo, São Paulo, SP – Brasil; 2 UPMC Children’s Hospital of Pittsburgh University of Pittsburgh School of Medicine Pittsburgh Pennsylvania EUA UPMC Children’s Hospital of Pittsburgh, University of Pittsburgh School of Medicine, Pittsburgh, Pennsylvania – EUA

**Keywords:** Síndrome do Coração Esquerdo Hipoplásico, Procedimento de Norwood, Oxigenação por Membrana Extracorpórea, Mortalidade

## Abstract

**Fundamento:**

Apenas dois artigos abordam os resultados precoces de pacientes com síndrome do coração esquerdo hipoplásico (SHCE) submetidos à operação de Norwood, no Brasil.

**Objetivos:**

Avaliamos pacientes com SHCE submetidos ao primeiro estágio da operação de Norwood para identificar os fatores preditivos de mortalidade precoce (nos primeiros 30 dias após a cirurgia) e intermediária (desde a sobrevida precoce até o procedimento de Glenn).

**Métodos:**

Foram incluídos pacientes com SHCE submetidos em nosso serviço ao primeiro estágio da operação de Norwood de janeiro de 2016 a abril de 2019. Dados demográficos, anatômicos e cirúrgicos foram analisados. Os desfechos foram mortalidade precoce (nos primeiros 30 dias após a cirurgia), mortalidade intermediária (desde a sobrevida precoce até o procedimento de Glenn) e a necessidade de suporte pós-operatório com ECMO. Foram realizadas análises univariadas e multivariadas e calculados odds ratios, com intervalos de confiança de 95%. Um valor de p < 0,05 foi considerado estatisticamente significativo.

**Resultados:**

Um total de 80 pacientes com SHCE foram submetidos ao primeiro estágio da operação de Norwood. A taxa de sobrevida em 30 dias foi de 91,3% e a taxa de sobrevida intermediária foi de 81,3%. Quatorze pacientes (17,5%) necessitaram de suporte com ECMO. Menor peso (p=0,033), estenose aórtica (vs atresia aórtica; p=0,036) e necessidade de suporte pós-operatório com ECMO (p=0,009) foram fatores preditivos independentes para mortalidade em 30 dias. A estenose da valva mitral ( *vs* atresia da valva mitral; p=0,041) foi um fator preditivo independente para mortalidade intermediária.

**Conclusão:**

O presente estudo inclui a maior coorte brasileira de pacientes com SHCE submetidos ao primeiro estágio da operação de Norwood na era recente. Nossas taxas de sobrevida foram comparáveis às mais altas taxas de sobrevida relatadas globalmente. Baixo peso corporal, estenose valvar aórtica e necessidade de suporte pós-operatório com ECMO foram preditores independentes para mortalidade em 30 dias. A estenose da valva mitral foi o único fator preditivo independente para mortalidade intermediária.

## Introdução

A síndrome do coração esquerdo hipoplásico (SHCE) é um defeito cardíaco congênito complexo que resulta em um coração subdesenvolvido com ventrículo esquerdo hipoplásico, valvas mitral e aórtica estenóticas ou atrésicas e hipoplasia da aorta ascendente e arco aórtico. A doença está associada a uma alta taxa de mortalidade e é atualmente tratada com uma estratégia de paliação cirúrgica em três estágios. No primeiro estágio, uma neoaorta é reconstruída e é criado um shunt sistêmico-pulmonar ou um conduto do ventrículo direito para a artéria pulmonar. No segundo estágio, é feita uma conexão cavopulmonar parcial (procedimento de Glenn) e, no terceiro estágio, uma conexão cavopulmonar total (procedimento de Fontan-Kreutzer).

A SHCE é quase sempre fatal sem paliação cirúrgica. No entanto, desde que Norwood descreveu pela primeira vez sua técnica de reconstrução paliativa de SHCE,^1^ as taxas de sobrevida aumentaram progressivamente.^2^ Atualmente, a taxa de sobrevida precoce é menor do que para outras cardiopatias congênitas, que requerem intervenção cirúrgica neonatal.^3^ Notavelmente, maior mortalidade ocorre no período entre os procedimentos de Norwood e Glenn, chegando perto de 25%.^4^ Muitos fatores diferentes podem contribuir para as taxas de sobrevida, incluindo peso corporal e idade na cirurgia, tamanho e função das valvas e câmaras cardíacas, tamanho da aorta nativa e variáveis intrínsecas ao procedimento cirúrgico (tempo de circulação extracorpórea (CEC), tamanho do shunt, e bandagem do shunt para controlar a taxa de fluxo pulmonar excessiva). A identificação desses fatores de risco pode contribuir para o aprimoramento dos conceitos gerais de tratamento, técnica cirúrgica e medidas terapêuticas auxiliares, a fim de melhorar as taxas de sobrevida.

Poucos relatos abordam os resultados precoces de pacientes com SHCE submetidos à operação de Norwood no Brasil.^7 , 8^ Esses relatos vêm de épocas anteriores e descrevem coortes de pacientes acumuladas por longos períodos de tempo. Aqui, objetivamos avaliar a sobrevida precoce (primeiros 30 dias de pós-operatório) e intermediária (período entre a sobrevida precoce e o procedimento de Shunt e Glenn) de pacientes com SHCE submetidos à operação de Norwood-Sano durante um período rigoroso de tempo (40 meses) na era do suporte de oxigenação por membrana extracorpórea (ECMO) e outros avanços médicos, em um centro de referência no Brasil. O objetivo do estudo foi identificar fatores preditivos para mortalidade pós-operatória precoce e intermediária, bem como para suporte pós-operatório de ECMO.

## Métodos

O presente estudo segue a declaração *Strengthening the Reporting of Observational Studies in Epidemiology* (STROBE).^9^ Foi avaliada uma coorte retrospectiva incluindo uma série sucessiva de pacientes, do setor privado ou público, diagnosticados com SHCE (Classificação Internacional de Doenças, 10° revisão, código Q23.4) e submetidos ao procedimento de Norwood por parte do nosso grupo no Hospital Beneficência Portuguesa de São Paulo, entre janeiro de 2016 e abril de 2019. Os critérios de exclusão incluíram pacientes sindrômicos, lactentes com hemorragia cerebral grave ou infarto, ou aqueles com complicações graves (por exemplo, suporte de ECMO) durante o período pré-operatório.

As variáveis independentes avaliadas neste estudo foram demográficas (idade, peso e sexo), anatômicas (tipo e tamanho da comunicação interatrial, presença de atresia aórtica e/ou mitral, diâmetro da aorta ascendente e tamanho da persistência do canal arterial), e cirúrgicas (diâmetro do shunt, bandagem do tubo Gore-Tex e tempos de CEC (pinçamento aórtico e parada circulatória total). Os dois objetivos principais foram determinar as taxas de sobrevida precoce (do pós-operatório imediato a 30 dias de cirurgia) e intermediária (de 30 dias até o procedimento de Glenn). Também investigamos a necessidade de suporte de ECMO.

Todos os dados clínicos e cirúrgicos desta coorte de pacientes foram recuperados do banco de dados institucional. O estudo foi aprovado pelo Comitê de Ética em Pesquisa da instituição.

### Manejo pré-operatório

Pacientes do setor privado vieram de todas as partes do Brasil e todas tinham diagnóstico fetal prévio. O parto foi realizado em nosso serviço e a paciente imediatamente transferida para nossa unidade de terapia intensiva cardíaca (UTIC). Normalmente, a cesárea é marcada para 38 ou 39 semanas de gestação, porém trabalho de parto normal também podia ocorrer de acordo com o desejo da família. Em nosso serviço ocorrem em média 50 partos por ano (dois partos SHCE/mês). Pacientes encaminhados de serviços públicos geralmente foram diagnosticados após o nascimento e internados o mais rápido possível.

Na UTIC, um cateter venoso umbilical é inserido e prostaglandina E1 em baixa dose (PGE_1_ 0,005-0,01mcg/kg/min) é iniciada para manter a permeabilidade ductal com baixo risco de apnéia. Se não houver necessidade de manipulação imediata do septo atrial, a cirurgia ocorre com 3-5 dias de vida. A técnica de preferência é a cirurgia de Norwood-Sano, conforme detalhado adiante. O débito cardíaco é monitorado por medidas clínicas e laboratoriais (débito urinário, perfusão periférica, pressão arterial, NIRS, gasometria arterial, lactato e saturação venosa central). Os lactentes clinicamente instáveis podem receber milrinona, epinefrina em baixa dose e mistura de gases hipóxicos, adicionando nitrogênio para reduzir a FiO_2_ até 17%, para tratar a síndrome do baixo débito cardíaco. Lactentes com apnéia secundária a PGE_1_ ou hemodinâmica instável persistente secundária à hipercirculação pulmonar geralmente se beneficiam de intubação endotraqueal e ventilação controlada antes da cirurgia.

### Técnica operatória

Abertura do tórax através de esternotomia mediana e um fragmento de pericárdio é retirado e tratado com glutaraldeído 0,6% por 30 minutos. A aorta ascendente, arco aórtico, canal arterial e aorta descendente proximal foram expostos. A circulação extracorpórea é estabelecida por canulação do canal arterial e do apêndice atrial direito. A cânula arterial foi levada através do canal arterial até a aorta descendente e um torniquete é apertado ao redor do canal e da cânula, o que permite que parte da operação fosse realizada sem parada circulatória. Enquanto o paciente está sendo resfriado, o ducto arterial é seccionado próximo à artéria pulmonar e seu coto proximal, saturado. A artéria pulmonar é então seccionada próxima à sua bifurcação, desconectando a artéria pulmonar distal e seus ramos pulmonares da artéria pulmonar principal. A abertura do coto distal da artéria pulmonar foi reduzida com uma pequena plicatura transversal, com a com a colocação de um ou dois pontos de Prolene 7.0 nas bordas da parede anterior e posterior. Em seguida, um conduto de politetrafluoretileno (PTFE) (geralmente 5 mm) é biselado para corresponder ao tamanho da abertura resultante na artéria pulmonar e suturado diretamente em seu coto distal, completando o preparo da artéria pulmonar distal. À medida que a temperatura esofágica foi gradualmente reduzida para 18ºC, pinçamos a aorta ascendente hipoplásica nativa distalmente. Em seguida, fazemos uma pequena incisão aórtica anterolateral longitudinal próximo ao local do pinçamento para introduzir uma agulha dobrável de ponta em forma de oliva em direção à artéria coronária. Esse instrumento especial, cujo tamanho atenderia ao diâmetro da aorta ascendente, serve para infundir a solução de cardioplegia Del Nido na aorta proximal. Às vezes, é necessário apertar a aorta ascendente ao redor da agulha com uma pinça para evitar a perda de cardioplegia. Alternativamente, um torniquete pode ser colocado ao redor da aorta para apertar suavemente a aorta ao redor da agulha de cardioplegia. Em seguida, estendemos essa incisão aórtica inicial longitudinalmente até perto da artéria coronária. A porção proximal da aorta ascendente é anastomosada na face lateral do tronco da artéria pulmonar com sutura contínua de Prolene 7.0, iniciando-se a reconstrução da neoaorta. Somente neste momento, a CEC é interrompida e a cânula arterial é retirada da parte distal do canal arterial. O tecido ductal remanescente é completamente excisado, e a abertura resultante estendeu-se proximalmente em direção ao arco aórtico e à aorta ascendente, bem como distalmente. Um enxerto de pericárdio autólogo tratado com glutaraldeído 0,6% foi utilizado para ampliação da aorta ascendente, do arco aórtico e da aorta descendente, que foi anastomosada ao tronco pulmonar, completando a neoaorta. Não são feitos testes para pontos de vazamento na linha anastomótica. A cânula arterial é novamente colocada no tronco pulmonar (neoaorta). A retirada de ar do coração e da aorta é realizada pelo enchimento lento da linha arterial, mantendo-se torniquetes aplicados nos ramos do arco, bem como uma pequena abertura na linha de sutura anterior da neoaorta proximal. A CEC é reiniciada com restabelecimento do fluxo e preparo para anastomose proximal do tubo VD-TP. A CEC é novamente interrompida por 2-3 minutos para ampliação da comunicação interatrial ou para uma anuloplastia tricúspide, quando necessário. A ampliação do septo atrial foi realizada através de uma atriotomia abaixo da bolsa da cânula venosa.

Para completar a circulação pulmonar, uma pequena incisão é feita na via de saída do VD e um orifício de aproximadamente 5 mm é confeccionado. Em seguida, o conduto PTFE que já estava anastomosado às artérias pulmonares passou a ser conectado a esse orifício. Para essa anastomose, utilizamos a técnica de sutura contínua de Prolene 6.0 que transpassa todas as camadas miocárdicas. Geralmente não biselamos o lado proximal do conduíte PTFE. Nenhuma cola cirúrgica é aplicada rotineiramente. Em geral, o batimento cardíaco retorna espontaneamente quando a CEC é reiniciada juntamente com o reaquecimento. O tórax é mantido aberto com uma membrana de látex suturada nas bordas da pele. Um adesivo plástico estéril foi aplicado sobre a membrana e a pele circundante para melhor isolamento da ferida. O fechamento esternal tardio geralmente é realizado em 24 a 48 horas, uma vez alcançada a estabilidade circulatória.

A cirurgia de Norwood-Sano foi utilizada na grande maioria dos casos, principalmente para prevenir a redução do fluxo coronariano durante a diástole, facilitando o manejo pós-operatório. Essa estratégia foi baseada nos resultados publicados anteriormente por nosso grupo, mostrando menor mortalidade nos pacientes submetidos a Norwood-Sano.^7^

Usamos a mesma técnica mesmo para aortas muito pequenas, mas, nesses casos, ampliamos a aorta nativa até mais próximo do plano ostial da artéria coronária, ajustando a anastomose com uma pequena incisão realizada no coto do tronco pulmonar (TP) proximal. Em alguns pacientes < 2,5Kg (n=2, 2,5%), a operação de Norwood foi adiada, e a bandagem cirúrgica seletiva de ambas as artérias pulmonares foi realizada, enquanto a infusão de prostaglandina foi mantida. Esses pacientes foram submetidos à operação de Norwood quando atingiram um peso alvo em tormo de 3 kg. A operação não foi contra-indicada a nenhum paciente e nem foram oferecidos apenas cuidados de suporte clínico.

Nos pacientes com peso corporal entre 2,5 e 2,7 kg, foi utilizado um conduto VD-TP de 4 mm. No pós-operatório, todos os pacientes com hemodinâmica instável associada a fluxo pulmonar excessivo foram tratados com bandagem do tubo VD-TP com fio absrovível Monocryl 5-0, estenosado a critério do cirurgião. No momento do fechamento do toráx, avalia-se a retirada da bandagem. Em pacientes com suspeita de alta resistência vascular pulmonar por encaminhamento tardio para tratamento cirúrgico ou comunicação interatrial restritiva, preferimos realizar o procedimento clássico de Norwood com shunt de Blalock-Taussig modificado de 3,5 ou 4,0 mm.

### Manejo pós-operatório

Todos os pacientes foram transferidos para a UTIC com o tórax aberto. O tórax foi geralmente fechado com 24-48h de pós- operatório, desde que a estabilidade hemodinâmica já tivesse sido alcançada. O suporte inotrópico e vasoativo foi realizado regularmente com milrinona e adrenalina e, se possível, associado à infusão contínua de Amplictyl (clorpromazina). Usamos um cateter de diálise peritoneal (DP) na maioria das crianças, mesmo naquelas com débito urinário adequado. A DP geralmente foi iniciada nos primeiros dias de pós-operatório com dialisato isotônico e/ou hipertônico para controlar a sobrecarga hídrica. Utilizamos suporte com ECMO em pacientes que evoluíram para síndrome refratária de baixo débito cardíaco (baixo débito urinário, hipotensão, alta necessidade de suporte inotrópico e/ou elevação de lactato), hipoxemia persistente, arritmias, parada cardíaca ou falha no desmame de CEC. A maioria dos pacientes foi colocada em suporte de ECMO na UTI, antes do fechamento do esterno. Em apenas dois pacientes (14,3%) foi iniciada ECMO no centro cirúrgico para desmame da CEC. A arritmia foi responsável pelo início da ECMO em apenas um paciente (7%). A assistência da ECMO sempre foi realizada por meio de canulação central e a ferida operatória foi mantida aberta até que a estabilização clínica permitisse a decanulação.

Devido ao referenciamento muitas vezes distante, adotamos uma política comum de manter todos os pacientes desta coorte internados até a recuperação do segundo estágio cirúrgico.

### Análise estatística

Os dados qualitativos foram descritos como frequências com porcentagens e os quantitativos como medianas com intervalos interquartis. Todos os dados foram tratados como não paramétricos devido ao tamanho da amostra. Para avaliar as associações entre os dados qualitativos, foi realizado o teste exato de Fisher. Para comparar dados quantitativos entre sobreviventes e não sobreviventes, foi utilizado o teste U de Mann-Whitney. Uma análise de sobrevida de Kaplan-Meier foi realizada e o teste log-rank foi usado para determinar diferenças significativas na sobrevida entre os estratos. A regressão logística foi realizada para identificar os preditores univariados e multivariados de mortalidade. Variáveis com p<0,25 na análise univariada foram incluídas na análise multivariada e o método *backward conditional stepwise* foi utilizado para definir o modelo final. Os resultados são apresentados como odds ratios com intervalos de confiança de 95% e valores de p. Um valor de p < 0,05 foi considerado estatisticamente significativo. Os dados foram analisados e plotados usando IBM SPSS Statistics para Windows (Versão 25.0; IBM Corp, Armonk, NY) e GraphPad Prism (Versão 6.01; GraphPad Software, Inc., La Jolla, Estados Unidos).

## Resultados

Um total de 80 pacientes com SHCE foram submetidos ao procedimento de Norwood (estágio I) entre janeiro de 2016 e abril de 2019. O procedimento de Norwood estágio I foi realizado em 80 pacientes (do sector privado, n=79, 98,7%; do sector público, n=1, 1,3%). O procedimento de Norwood-Sano foi realizado em 78 (97,5%) pacientes e o Norwood clássico, em 2 (2,5%). A taxa de sobrevida precoce da coorte total foi de 91,3% (n=73), enquanto a taxa de sobrevida intermediária foi de 81,3% (n=65).

### Demografia

Cinquenta e um pacientes (63,8%) foram do sexo masculino, a idade mediana na cirurgia foi de 3,0 (1,0-147,0) dias e o peso médio foi de 3.080 (2.765-3.360) gramas. Os dados estratificados para sobreviventes (73 pacientes) e não sobreviventes (7 pacientes), bem como as comparações entre os grupos, estão descritos na Tabela 1 . Resumidamente, os não sobreviventes de 30 dias de pós-operatório apresentaram menor peso no momento da cirurgia (p=0,0257). Não foram encontradas diferenças para as demais características demográficas.

**Tabela 1:** Características demográficas

Sexo (masculino)	Sobreviventes	Não sobreviventes (30 dias)	valor p
	46/73 (63,0%)	5/7 (71,4%)	1,000
**Peso (g)**	**Sobreviventes**	**Não sobreviventes (30 dias)**	**valor p**
	3115 (2820-3440)	2740 (2500-2990)	0,0257
**Idade(dias)**	**Sobreviventes**	**Não sobreviventes (30 dias)**	**valor p**
	3,0 (1,0-147,0)	3,0 (2,0-5,0)	0,1893

### Anatomia

As características anatômicas foram descritas em relação ao tamanho da comunicação interatrial, anatomia das valvas mitral e aórtica, tamanho da aorta ascendente e persistência do canal arterial. Para pacientes com um único defeito do septo atrial (DSA), o tamanho médio do defeito foi de 3,55 (2,65-4,73) mm. Para pacientes com múltiplos defeitos do septo atrial, a área total estimada do DSA foi de 10,8 (6,1-18,1) mm^2^. A valva mitral estava normal em 1,3% (n=1) dos pacientes, estenótica em 53,7% (n=43), atrésica em 43,7% (n=35) e um caso apresentava valva atrioventricular única. A valva aórtica estava normal em 2,5% dos pacientes (n=2), estenótica em 30% (n=24) e atrésica em 67,5% (n=54). O tamanho da aorta ascendente foi de 2,7 (2,0-4,3) mm e o tamanho da persistência do canal arterial foi de 5,8 (5,00-6,5) mm. As variáveis anatômicas dos grupos de pacientes sobreviventes e não sobreviventes foram semelhantes ( Tabela 2 ). Não encontramos diferenças significativas em relação à anatomia dos pacientes.

**Tabela 2:** Características anatômicas

DSA			
Área (mm^2^)	Sobreviventes	Não sobreviventes (30 dias)	valor p
área total estimada do DSA	10,95 (5,93-18,10)	8,7 (7,10-20,40)	0,7714
**Válvulas do Coração (Esquerda)**			
**Valva mitral**	**Sobreviventes**	**Não sobreviventes (30 dias)**	**valor p**
normal	1/72 (1,4%)	0/7 (0,0%)	0,2187
estenose	37/72 (51,4%)	6/7 (85,7%)	
atresia	34/72 (47,2%)	1/7 (14,3%)	
**Valva aórtica**	**Sobreviventes**	**Não sobreviventes (30 dias)**	**valor p**
normal	2/73 (2,7%)	0/7 (0,0%)	0,2508
estenose	20/73 (27,4%)	4/7 (57,1%)	
atresia	51/73 (69,9%)	3/7 (42,9%)	
**Subgrupos**	**Sobreviventes**	**Não sobreviventes (30 dias)**	**valor p**
MS/AS	17/73 (23,3%)	4/7 (57,1%)	0,3267
MS/AA	19/73 (26,0%)	2/7 (28,6%)	
MA/AS	2/73 (2,7%)	0/7 (0,0%)	
MA/AA	32/73 (43,8%)	1/7 (14,3%)	
outro	3/73 (4,1%)	0/7 (0,0%)	
**Aorta**			
**Aorta ascendente**	**Sobreviventes**	**Não sobreviventes (30 dias)**	**valor p**
tamanho (mm)	2,80 (2,00-4,30)	2,00 (2,00-6,20)	0,6612
**Persistência do canal arterial**	**Sobreviventes**	**Não sobreviventes (30 dias)**	**valor p**
tamanho (mm)	5,75 (5,00-6,50)	6,70 (4,50-7,30)	0,5569

*NA: não aplicável. DSA: defeito do septo atrial.*

### Dados cirúrgicos

O diâmetro do shunt nos dois pacientes submetidos à operação clássica de Norwood foi de 3,5 mm. Naqueles que foram submetidos à operação de Norwood-Sano, foi selecionado um enxerto de 4,0 mm (n=21; 27%) ou 5,0 mm (n=57; 73%). Em 25 (32,4%) pacientes foi utilizada bandagem de shunt. Os tempos medianos de CEC, pinçamento aórtico e parada circulatória foram, respectivamente, 188 (170-214) min, 76 (70-80) min e 48 (45-53) min. Não foi encontrada diferença significativa entre os grupos de pacientes sobreviventes e não sobreviventes ( Tabela 3 ).

**Tabela 3:** Cirurgia

Diâmetro da derivação (mm)	Sobreviventes	Não sobreviventes (30 dias)	valor p
3.5	1/41 (2.4%)	0/3 (0.0%)	0.9403
4.0	11/41 (26.8%)	1/3 (33.3%)	
5.0	29/41 (70.7%)	2/3 (66.7%)	
**Faixas**	**Sobreviventes**	**Não sobreviventes (30 dias)**	**valor p**
pacientes submetidos a bandagem do shunt	11/32 (34,4%)	0/2 (0%)	0,3134
**Horários da cirurgia**	**Sobreviventes**	**Não sobreviventes (30 dias)**	**valor p**
CEC (min)	185 (170-210)	205 (180-240)	0,2202
Pinçamento aórtico (min)	76 (70-80)	77 (59-82)	0,7057

*Obs.: Faltaram dados para as variáveis “tamanho do shunt” e “bandagem do shunt” para 36 e 44 pacientes respectivamente. Por esse motivo, não utilizamos essas variáveis na análise uni ou multivariada. CEC: circulação extracorpórea.*

### Mortalidade precoce e intermediária e suporte com ECMO

**Figura 1:**
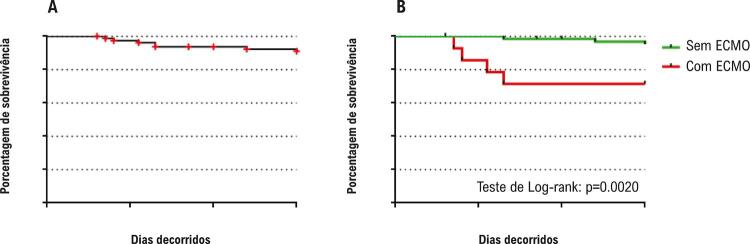
Taxas de sobrevida precoce (até 30 dias pós-operatórios) após o procedimento de Norwood estágio I. A) Coorte inteira (n=80). B) Comparação entre pacientes com ECMO (n=14) vs sem ECMO (n=66). ECMO: oxigenação por membrana extracorpórea.

Nos primeiros 30 dias de pós-operatório, 7 pacientes (8,7%) morreram, resultando em uma taxa de sobrevida precoce de 91,3% ( Figura 1A ). Além disso, durante esses primeiros 30 dias, 14 pacientes (17,5%) necessitaram de suporte com ECMO. Entre os 73 pacientes que sobreviveram, apenas 13,7% receberam ECMO, contra 57,1% dos 7 não sobreviventes(p=0,0039). As curvas de sobrevida estratificada para pacientes que necessitam ou não de ECMO estão ilustradas na Figura 1B . A comparação das curvas de sobrevida indica pior resultado para aqueles que necessitaram de suporte circulatório (teste Log-rank, p=0,0020).

**Figura 2:**
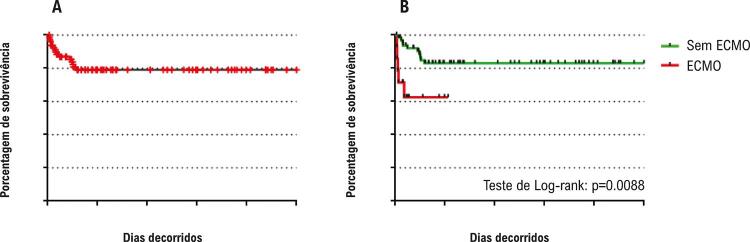
Taxas de sobrevida intermediárias (de 30 dias de pós-operatório até o procedimento de Glenn). A) Coorte inteira (n=73). B) Comparação entre pacientes com ECMO (n=9) vs sem ECMO (n=64). ECMO: oxigenação por membrana extracorpórea.

A taxa de sobrevida intermediária foi de 81,3%, uma vez que 8 pacientes adicionais morreram entre o 30º dia de pós-operatório e o procedimento de Glenn ( Fig.2A ). A ECMO foi empregada em 33,3% (n=3) dos 8 não sobreviventes, e em 13,8% (n=9) dos que receberam a operação de Glenn. A Figura 2B mostra que os pacientes suportados pela ECMO apresentaram pior resultado em relação aos que não necessitaram de ECMO (teste Log-rank, p=0,0088).

Lesão neurológica irreversível ocorreu em 4 crianças (28%) submetidas ao tratamento com ECMO. A diálise foi necessária em 85% (n=68) dos casos. Todos os sobreviventes recuperaram a função renal. A coarctação pós-operatória da aorta exigiu reintervenção em 6 (7,5%) pacientes (implante percutâneo de stent, n=4; ampliação cirúrgica simultânea ao procedimento de Glenn, n= 2).

### Preditores de assistência com ECMO, mortalidade cirúrgica em 30 dias e mortalidade intermediária

As Tabelas 4 a 6 exploram os potenciais preditores para assistência com ECMO, mortalidade precoce e intermediária, respectivamente. Pela análise univariada, o tempo de CEC foi único fator preditivo para assistência com ECMO, pois nenhuma outra variável atingiu o limiar de p< 0,250 ( Tabela 4 ). Por esse motivo, não foi possível realizar uma análise multivariada e nenhuma variável pôde ser confirmada como preditora independente de ECMO.

**Tabela 4:** Preditores de assistência com ECMO segundo regressão logística univariada e multivariada

	univariada		multivariada	

	OR (95%CI)	valor p	OR (95%CI)	valor p
**Demografia**				
Sexo (para homens)	1,524 (0,432-5,383)	0,513	-	-
Peso (por g)	1,000 (0,998-1,001)	0,515	-	-
Idade (por dia)	0,860 (0,606-1,220)	0,397	-	-
**Anatomia**				
área total estimada de DSA (por mm^2^)	0,982 (0,944-1,021)	0,354	-	-
Valva mitral (atresia vs. estenose)	0,905 (0,282-2,909)	0,867	-	-
Valva aórtica (atresia vs. estenose)	1,791 (0,451-7,112)	0,408	-	-
Tamanho Asc Ao (por mm)	0,907 (0,635-1,295)	0,590	-	-
**Cirurgia**				
CEC (por minuto)	1,006 (0,996-1,016)	0,248	1,006 (0,996-1,016)	0,248
Pinçamento aórtico (por min)	0,997 (0,944-1,054)	0,922	-	-

*DSA: defeito do septo atrial; CEC: circulação extracorpórea.*

Em relação à mortalidade pós-operatória em 30 dias ( Tabela 5 ), peso corporal, anatomia das valvas mitral e aórtica, tempo de CEC e assistência com ECMO foram considerados fatores preditivos pela análise univariada. No entanto, pela análise multivariada, a anatomia da valva mitral e o tempo de CEC não se mantiveram como preditores independentes, em contraste com o peso corporal, a anatomia da valva aórtica e a necessidade de suporte com ECMO, que se confirmaram como fatores de risco independentes. Quanto maior o peso, menor o risco (OR 0,997 por grama; CI 95% 0,995-1,000; p=0,033). A atresia da valva aórtica foi fator protetor em relação à estenose (OR 0,090; CI 95% 0,009-0,857; p=0,036), e a necessidade de ECMO pós-operatória foi um importante fator de risco independente para mortalidade (OR 20,975; CI 95% 2,116-207,886; p=0,009). O tamanho do shunt e a bandagem do tubo Sano não puderam ser analisados por regressão logística uni/multivariada devido à falta de dados.

**Tabela 5:** Preditores de mortalidade em 30 dias segundo regressão logística univariada e multivariada

	univariada		multivariada	

	OR (95%CI)	valor p	OR (95%CI)	valor p
**Demografia**				
Sexo (para homens)	1,467 (0,266-8,091)	0,660	-	-
Peso (por g)	0,998 (0,996-1,000)	0,056	0,997 (0,995-1,000)	0,033
Idade (por dia)	0,734 (0,382-1,413)	0,355	-	-
**Anatomia**				
área total estimada de DSA (por mm^2^)	1,010 (0,983-1,037)	0,465	-	-
válvula mitral (atresia vs. estenose)	0,181 (0,021-1,585)	0,123	0,491 (0,024-10,089)	0,645
válvula aórtica (atresia vs. estenose)	0,294 (0,060-1,433)	0,130	0,090 (0,009-0,857)	0,036
Tamanho Asc Ao (por mm)	0,981 (0,620-1,552)	0,935	-	-
**Cirurgia**				
CEC (por minuto)	1,010 (0,999-1,021)	0,089	1,018 (0,992-1,044)	0,173
Pinçamento aórtico (por min)	0,952 (0,874-1,037)	0,260	-	-
ECMO	8,400 (1,631-43,256)	0,011	20,975 (2,116-207,886)	0,009

*DSA: defeito do septo atrial; CEC: circulação extracorpórea; ECMO: oxigenação por membrana extracorpórea.*

Pela análise univariada, anatomia das valvas mitral e aórtica, tempo de CEC e suporte com ECMO foram fatores preditivos de mortalidade intermediária ( Tabela 6 ). Na análise multivariada, porém, a anatomia da valva mitral surgiu como o único preditor de mortalidade. A estenose valvar levou a um pior prognóstico em relação à atresia valvar (OR 0,242; 95% CCI 0,062-0,942; p=0,041).

**Tabela 6:** Preditores intermediários de mortalidade segundo regressão logística univariada e multivariada

	univariada		multivariada	

	OR (95%CI)	valor p	OR (95%CI)	valor p
**Demografia**				
Sexo (para homens)	1,719 (0,493-5,991)	0,395	-	-
Peso (por g)	1,000 (0,998-1,001)	0,511	-	-
Idade (por dia)	1,008 (0,986-1,031)	0,457	-	-
**Anatomia**				
Área total estimada de DSA (por mm^2^)	1,010 (0,989-1,031)	0,354	-	-
Valva mitral (atresia vs. estenose)	0,242 (0,062-0,942)	0,041	0,242 (0,062-0,942)	0,041
Valva aórtica (atresia vs. estenose)	0,422 (0,133-1,343)	0,144	0,357 (0,059-2,174)	0,264
Tamanho Asc Ao (por mm)	1,003 (0,723-1,392)	0,984	-	-
**Cirurgia**				
CEC (por minuto)	1,008 (0,998-1,018)	0,131	1,017 (0,998-1,037)	0,080
Pinçamento aórtico (por min)	0,992 (0,936-1,051)	0,785	-	-
ECMO	3,111 (0,862-11,231)	0,083	3,011 (0,623-14,542)	0,170

*DSA: defeito do septo atrial; CEC: circulação extracorpórea; ECMO: oxigenação por membrana extracorpórea.*

## Discussão

Desde o estabelecimento dos procedimentos clássicos de Norwood ou Norwood-Sano como tratamento cirúrgico padrão para o tratamento de pacientes com SHCE, houve uma melhora progressiva em todo o mundo na taxa de sobrevida. O presente estudo incluiu 80 pacientes consecutivos operados a partir de 2016 que foram extraídos de nossa série de mais de 500 pacientes para representar os resultados iniciais atuais na era do suporte com ECMO. Nossa taxa de mortalidade precoce de 8,7% para pacientes submetidos aos procedimentos de Norwood/Norwood-Sano está entre as mais baixas relatadas.^10 , 11^ Outros relataram uma mortalidade pós-operatória em 30 dias de 15,2%.^12^ A mortalidade provisória (depois da alta hospitalar após o procedimento de Norwood até a operação de Glenn) varia de 5-28%.^13^ De acordo com o Banco de Dados de Cirurgia Cardíaca Congênita da *Society of Thoracic Surgeons* (STS’s),^20^ a mortalidade geral é de 22%, enquanto a mortalidade para pacientes com qualquer complicação (27%) é muito maior (p<0,0001) em comparação aos pacientes que não sofreram complicações (7%).

No presente estudo, o baixo peso corporal foi encontrado como preditor independente de mortalidade precoce após a operação de Norwood estágio I, de acordo com o descrito por vários estudos anteriores.^12 , 16 , 21 , 22^

A anatomia das valvas aórtica e mitral também foi reconhecida como preditora de mortalidade precoce em estudos anteriores.^23 - 29^ A presença de atresia aórtica e/ou mitral geralmente está associada a maiores taxas de mortalidade, principalmente quando a estenose mitral é acompanhada de atresia aórtica,^23 , 24 , 29^ ou está associada a uma CIA restritiva.^25^ Na presente investigação, tanto a anatomia da valva aórtica quanto a necessidade de assistência com ECMO mostraram-se como fatores de risco independentes para mortalidade precoce. Curiosamente, em nosso estudo, a atresia valvar aórtica, em comparação com a estenose valvar aórtica, foi fator protetor contra a mortalidade, e o mesmo ocorreu em relação à anatomia valvar mitral.

Além disso, detectamos a necessidade de suporte com ECMO como um importante fator de risco independente para mortalidade precoce. De fato, os pacientes com ECMO tiveram um risco de mortalidade 20 vezes maior do que os pacientes que não precisaram de suporte circulatório mecânico. Infelizmente, não conseguimos isolar nenhum fator preditivo independente para assistência com ECMO, embora estudos anteriores tenham relatado peso ao nascer <2,5 kg e maior tempo de CEC como independentemente associados à necessidade de ECMO após a operação de Norwood.^30^

No presente estudo, a atresia valvar mitral e o tempo prolongado de CEC apareceram como preditores de mortalidade precoce pela análise univariada, mas não foram confirmados como preditores independentes de mortalidade precoce pela análise multivariada. Quando a mortalidade intermediária foi examinada, apenas a anatomia da valva mitral apareceu como fator de risco independente, estando a estenose da valva mitral correlacionada com pior prognóstico em relação à atresia valvar. Tempos prolongados de CEC não foram relatados como preditores de mortalidade no procedimento de Norwood,^14 , 16 , 18^ embora alguns estudos tenham relatado valores de p limítrofes.

Dois outros importantes preditores de mortalidade da operação de Norwood estágio I são os volumes cirúrgicos do centro e do cirurgião. Ambas as variáveis foram significativamente associadas aos resultados após o procedimento de Norwood de acordo com o banco de dados de cirurgia cardíaca congênita da STS.^31^ A STS relatou que centros que operam mais de 20 casos por ano e cirurgiões que operam mais de 10 casos por ano apresentam menores taxas de mortalidade. O presente estudo confirma o mesmo fora da América do Norte, já que as baixas taxas de mortalidade aqui relatadas derivam tanto do alto número de casos do centro quanto do cirurgião.

### Limitações

Reconhecemos que nosso estudo tem algumas limitações. As análises retrospectivas levaram a perda de dados, pois o registro de informações clínicas estava sendo migrado de manual para digital ao longo do período do estudo. Os resultados podem ter sido afetados negativamente, uma vez que a indicação de ECMO foi mais conservadora e às vezes atrasada no início da série, quando uma equipe interna de ECMO ainda não estava disponível. Consequentemente, o período entre a indicação de ECMO e o início do suporte de ECMO foi muito reduzido mais tarde na série.

## Conclusão

O presente estudo relata uma grande coorte brasileira de pacientes com SHCE submetidos ao procedimento de Norwood na era recente. Tivemos uma taxa de sobrevida em 30 dias de 91,3%, que é comparável às maiores taxas de sobrevida relatadas em todo o mundo e uma taxa de sobrevida intermediária de 81,3%. Baixo peso corporal, estenose aórtica (em comparação com atresia aórtica) e necessidade de suporte com ECMO foram preditores independentes de mortalidade em 30 dias, enquanto anatomia valvar mitral e aórtica, tempo de CEC e suporte com ECMO foram fatores preditivos de mortalidade intermediária. Nenhum fator de risco independente para suporte com ECMO pode ser evidenciado. Estudos futuros visando a mortalidade entre estágios, bem como a mortalidade de outros procedimentos envolvidos na reconstrução paliativa da SHCE, podem fornecer evidências adicionais para a taxa de sobrevida em longo prazo e adicionar outros potenciais fatores preditivos de mortalidade.
