# Supplementary material for: Resultados Precoces do Procedimento de Norwood em um Centro de Referência no Brasil
Source: Arq Bras Cardiol. 2022 Jun 6;119(2):282–91. [Article in Portuguese] doi: 10.36660/abc.20201226 (PMC9363068; doi:10.36660/abc.20201226)
Supplement: Translation

# Translation: Early Outcomes of the Norwood Procedure in a Reference Center in Brazil

**Authors:** Rodrigo Freire Bezerra, Juliana Torres Pacheco, Sônia Meiken Franchi, Rosangela Belbuche Fittaroni, José Francisco Baumgratz, Rodrigo Moreira Castro, Luciana da Fonseca da Silva, José Pedro da Silva

**Affiliations:** 1 Brazil Hospital Beneficência Portuguesa de São Paulo, São Paulo, SP – Brazil; 2 USA UPMC Children’s Hospital of Pittsburgh, University of Pittsburgh School of Medicine, Pittsburgh, Pennsylvania – USA

**Keywords:** Hypoplastic Left Heart Syndrome, Norwood Procedures, Extracorporeal Membrane Oxygenation, Mortality

## Abstract

**Background:**

Only two papers have addressed the early outcomes of patients with hypoplastic left heart syndrome (HLHS) undergoing the Norwood operation, in Brazil.

**Objectives:**

We evaluated patients with HLHS undergoing the first-stage Norwood operation in order to identify the predictive factors for early (within the first 30 days after surgery) and intermediate (from early survival up to the Glenn procedure) mortality.

**Methods:**

Patients with HLHS undergoing the stage I Norwood procedure from January 2016 through April 2019, in our service, were enrolled. Demographic, anatomical, and surgical data were analyzed. Endpoints were early mortality (within the first 30 days after surgery), intermediate mortality (from early survival up to the Glenn procedure) and the need for postoperative ECMO support. Univariate and multivariate analyses were performed, and odds ratios, with 95% confidence intervals, were calculated. A p-value <0.05 was considered statistically significant.

**Results:**

A total of 80 patients with HLHS underwent the stage I Norwood procedure. The 30-day survival rate was 91.3% and the intermediate survival rate 81.3%. Fourteen patients (17.5%) required ECMO support. Lower weight (p=0.033), aortic stenosis (vs aortic atresia; p=0.036), and the need for postoperative ECMO support (p=0.009) were independent predictive factors for 30-day mortality. Mitral valve stenosis (vs mitral valve atresia; p=0.041) was an independent predictive factor for intermediate mortality.

**Conclusion:**

The present study includes the largest Brazilian cohort of patients with HLHS undergoing the stage I Norwood procedure in the recent era. Our survival rates were comparable to the highest survival rates reported globally. Low body weight, aortic valve stenosis, and the need for postoperative ECMO support were independent predictors for 30-day mortality. Mitral valve stenosis was the only independent predictive factor for intermediate mortality.

## Introduction

Hypoplastic left heart syndrome (HLHS) is a complex congenital heart defect that results in an underdeveloped heart with a hypoplastic left ventricle, stenotic or atretic mitral and aortic valves, and hypoplasia of the ascending aorta and aortic arch. The disease is associated with a high mortality rate and currently treated with a three-stage surgical palliation strategy. At the first stage, a neoaorta is reconstructed and either a systemic-to-pulmonary shunt or right ventricle to pulmonary artery conduit created. During the second stage, a partial cavopulmonary connection is constructed (Glenn procedure) and, at the third stage, a total cavopulmonary connection is completed (Fontan-Kreutzer procedure).

The HLHS is nearly always fatal without surgical palliation. However, since Norwood first described his technique for the palliative reconstruction of HLHS,^1^ survival rates have progressively increased.^2^ The early survival rate is currently lower than for other congenital heart defects, which require neonatal surgical intervention.^3^ Notably, higher mortality occurs in the interstage period between the Norwood and Glenn procedures, reaching close to 25%.^4^ Many different factors may contribute to the survival rates, including body weight and age at surgery, size and function of the valves and heart chambers, native aorta size, and variables intrinsic to the surgical procedure (time under cardiopulmonary bypass [CPB], shunt size, and shunt banding in order to manage excessive pulmonary flow rate). The identification of such risk factors could contribute to the improvement in the general treatment concepts, surgical technique, and ancillary therapeutic measures, in order to improve the survival rates.

Few reports have addressed the early outcomes of patients with HLHS undergoing Norwood operation in Brazil.^7 , 8^ These reports came from previous eras and describe patient cohorts accumulated over long time periods. Here, we aim to evaluate the early (first 30 postoperative days) and intermediate (interstice between the early survival and the Glenn shunt procedure) survival of patients with HLHS undergoing the Norwood-Sano operation during a strict period of time (40 months) in the era of extracorporeal membrane oxygenation (ECMO) support and other medical advances, in a reference center in Brazil. The aim of the study was to identify predictive factors for early and intermediate post-operative mortalities, as well as for post-operative ECMO support.

## Methods

The current study follows the Strengthening the Reporting of Observational Studies in Epidemiology (STROBE) statement.^9^ A retrospective cohort including all successive patients, private or public, diagnosed with HLHS (International Classification of Diseases, 10^th^revision, code q23.4) and undergoing the Norwood procedure by our group at *Hospital Beneficência Portuguesa de São Paulo,* between January 2016 and April 2019, was evaluated. Exclusion criteria included syndromic patients, infants with severe cerebral hemorrhage or infarction, or those with severe complications (e.g ECMO support) during the preoperative period.

The independent variables evaluated in this study were demographic (age, weight, and sex), anatomical (type and size of atrial septal defect, presence of aortic and/or mitral atresia, ascending aorta diameter, and size of the patent ductus arteriosus), and surgical (shunt diameter, Gore-Tex tube banding, and CPB, cross-clamp, and cardiac arrest times). The two main objectives were to determine the early (30-day post-operative) and the intermediate (from 30-day post-operative throughout the Glenn procedure) survival rates. We also investigated the need for ECMO support.

All clinical and surgical data on this patient cohort were retrieved from the institutional database. The study was approved by the Research Ethics Committee of the institution.

### Preoperative management

Private patients came from all parts of Brazil and all of them had previous fetal diagnosis. The delivery was done in our service and the patient immediately transferred to our cardiac intensive care unit (CICU). Usually, a C-section is scheduled at 38 or 39 gestational weeks; however, normal labor may also occur according to the family’s desire. A mean of 50 deliveries per year take place in our service (two HLHS births/month). Patients referred from public services were usually diagnosed after birth and were admitted as soon as possible.

In the CICU, an umbilical venous catheter is inserted and low-dose prostaglandin E_1_(PGE_1_0.005-0.01mcg/kg/min) is initiated to maintain ductal patency with low risk of apnea. If there is no need for immediate atrial septum manipulation, surgery occurs at 3-5 days of life. The technique of preference is the Norwood-Sano surgery, as detailed ahead. Cardiac output is monitored using clinical and laboratorial measures (urine output, peripheral perfusion, blood pressure, NIRS, arterial blood gas, lactate and central venous saturation). The clinically unstable infants may receive milrinone, low dose epinephrine and hypoxic gas mixture by adding nitrogen to lower FiO_2_to 17%, in order to treat low cardiac output syndrome. Infants with apnea secondary to PGE_1_or persistent unstable hemodynamics secondary to pulmonary overcirculation usually benefit from endotracheal intubation and controlled ventilation before surgery.

### Operative technique

We entered the chest via median sternotomy and harvested a piece of pericardium, which was treated with glutaraldehyde 0.6% for 30 minutes. The ascending aorta, aortic arch, ductus arteriosus, and proximal descending aorta were exposed. Cardiopulmonary bypass was established by cannulation of the ductus arteriosus and the right atrial appendage. The arterial cannula was advanced through the ductus arteriosus into the descending aorta and a tourniquet tightened around the ductus and the cannula, which allows for part of the operation to be performed without circulatory arrest. While the patient was being cooled, the arterial ductus was divided near the pulmonary artery and its proximal stump sutured. The pulmonary artery was then divided close to its bifurcation, thus disconnecting the distal pulmonary artery and its pulmonary branches from the main pulmonary artery. The pulmonary artery distal stump opening was reduced with a small transverse plication, by placing one or two interrupted 7.0 Prolene sutures at its anterior and posterior wall edges. Next, a polytetrafluoroethylene (PTFE) conduit (usually 5 mm) was beveled to match the size of the resulting opening in the pulmonary artery and sutured directly to its distal stump, completing the distal pulmonary artery preparation. As the esophageal temperature was gradually reduced to 18ºC, we cross-clamped the native hypoplastic ascending aorta distally. Then, we made a small longitudinal anterolateral aortic incision near the clamped site to introduce an olive tip bendable needle toward the coronary artery. This special instrument, whose size would meet the ascending aorta diameter, serves to infuse the Del Nido cardioplegia solution into the proximal aorta. Sometimes, it is necessary to tighten the ascending aorta around the needle by pinching the aorta with a forceps to prevent cardioplegia wasting. Alternatively, a tourniquet can be placed around the aorta to gently tighten the aorta around the cardioplegia needle. Next, we extend that initial aortic incisionlongitudinally to near the coronary artery. The proximal portion of the ascending aorta was anastomosed on the lateral surface of the pulmonary artery trunk with 7.0 Prolene continuous suture, starting the neoaorta reconstruction. Only at this point, the CPB was interrupted, and the arterial cannula removed from the distal part of the ductus arteriosus. The remaining ductal tissue was completely excised, and the resulting opening extended proximally towards the aortic arch and ascending aorta, as well as distally. A 0.6% glutaraldehyde-treated autologous pericardial graft was used to enlarge the ascending aorta, aortic arch, and descending aorta, which was anastomosed to the pulmonary trunk, completing the neoaorta. No tests for leakings spots on the long anastomotic line were done. The arterial cannula was again placed in the pulmonary trunk (neoaorta). Aorta deairing was accomplished by slowly flushing the arterial line while keeping tourniquets applied to the aortic arch branches, as well as a small opening in the proximal neoaorta anterior suture line. The CPB was restarted, but rewarming was not yet initiated. The CPB was interrupted again for 2-3 minutes for an enlarged atrial septal defect or tricuspid annuloplasty when necessary. The atrial septal enlargement was performed through an atriotomy below the venous cannula purse string.

To complete the pulmonary circulation, a small incision is made in the RV outflow tract and a 5mm RV hole is punched out. Then, the PTFE conduit that was already anastomosed to the pulmonary arteries is now connected to that hole. For that anastomosis, we use a 6.0 Prolene running suture technique that trespasses all the myocardial layers. We usually don’t bevel the proximal side of the PTFE conduit. No surgical glue is routinely applied. In general, the heartbeat returns spontaneously as CPB is restarted and rewarming is initiated. The thorax was kept open using a latex membrane sutured to the skin edges. A sterile plastic adhesive was applied over the membrane and surrounding skin for better wound insulation. The delayed sternal closure was usually performed within 24 to 48 hours; once circulatory stability was ac hieved.

Norwood-Sano is used in the vast majority of cases, primarily to prevent reduction of coronary flow during diastole, thus facilitating postoperative management. This strategy was based on the results published previously by our group, showing lower mortality in patients undergoing Norwood-Sano.^7^

We used the same technique even for very small aortas but, in these cases, we enlarged the native aorta until closer to the coronary artery ostial plane, adjusting the anastomosis with a small incision performed in the proximal PA stump. In some < 2.5Kg patients (n=2, 2.5%), the Norwood operation was postponed, and surgical selective banding of both pulmonary arteries was otherwise performed, while the prostaglandin infusion was maintained. These patients were submitted to the Norwood operation when they reached a targeted weight short of 3 kg. No patient was denied operation and offered just clinical supportive care instead.

In patients with body weight between 2.5 and 2.7 kg, we used a 4-mm RV-PA conduit. In the post-operative period, all patients with unstable hemodynamics associated with excessive pulmonary flow were managed by banding the RV-PA conduit with a 5-0 Monocryl absorbable suture tied at the surgeon’s discretion. When the chest was closed, removal of the prolene suture band was considered, but we almost always ended up leaving it alone. In patients suspected of high pulmonary vascular resistance due to either late referral for surgical treatment or restrictive atrial septal defect, we preferred to perform the classic Norwood procedure with a 3.5 or 4.0-mm modified Blalock-Taussig shunt.

### Postoperative management

All patients were transferred to CICU with the chest left open. The chest was usually closed 24-48h postoperatively, provided that hemodynamic stability had already been achieved. Inotropic and vasoactive support was regularly achieved with milrinone and adrenaline and, if possible, associated with continuous infusion of Amplictyl (chlorpromazine). We used a peritoneal dialysis (PD) catheter in most children, even those with adequate urine output. PD was usually initiated in the first postoperative days with isotonic and/or hypertonic dialysate to manage fluid overload. We used ECMO support on patients who evolved to refractory low cardiac output syndrome (low urinary output, hypotension, high lactate and/or high inotropic requirements), persistent hypoxemia, arrhythmias, cardiac arrest, or failure to wean from cardiopulmonary bypass (CPB). Most patients were placed on ECMO support in the ICU, before the sternum was closed. In just two patients (14.3%), ECMO was started in the OR in order to wean them from CPB. Arrhythmia was responsible for ECMO initiation in just one patient (7%). ECMO assistance was always performed through central cannulation and the chest incision was kept open until clinical stabilization allowed for ECMO decannulation.

Due to the frequent distant referencing, we adopted a common policy of keeping all patients in this cohort hospitalized until they recovered from the second surgical stage.

### Statistical analysis

Qualitative data were described as frequencies with percentages, and quantitative data as medians with interquartile ranges. All data were treated as non-parametric due to the size of the sample. To evaluate associations between qualitative data, we performed Fisher’s exact test. To compare quantitative data among survivors and non-survivors, we used Mann–Whitney U test. A Kaplan-Meier survival analysis was performed, and the log-rank test was used to determine significant differences in survival between strata. Logistic regression was performed to identify the univariate and multivariate predictors of mortality. Variables with p<0.25 in the univariate analysis were included in the multivariate analysis and the backward conditional stepwise method used to define the final model. Results are presented as odds ratios with 95% confidence intervals and p-values. A p-value <0.05 was considered statistically significant. Data were analyzed and plotted using IBM SPSS Statistics for Windows (Version 25.0; IBM Corp, Armonk, NY) and GraphPad Prism (Version 6.01; GraphPad Software, Inc., La Jolla, United States).

## Results

A total of 80 patients with HLHS underwent the Norwood procedure (stage I) between January 2016 and April 2019. The stage I Norwood procedure was performed in 80 (private, n=79, 98.7%; public, n=1, 1.3%) patients. A Norwood-Sano procedure was performed in 78 (97,5%) patients, and a classic Norwood, in 2 (2.5%). The whole cohort early survival rate was 91.3% (n=73), while the intermediate survival rate was 81.3% (n=65).

### Demographics

Fifty-one patients (63.8%) were male, the median age at surgery was 3.0 (1.0-147.0) days, and the mean weight was 3080 (2765-3360) grams. The stratified data for survivors (73 patients) and non-survivors (7 patients), as well as the comparisons between the groups, are described in Table 1 . Briefly, 30 postoperative day non-survivors presented with a lower weight at the time of surgery (p=0.0257). No differences were found for the other demographic characteristics.

**Table 1:** Demographic characteristics

Sex (males)	Survivors	Nonsurvivors (30-day)	p-value
	46/73 (63.0%)	5/7 (71.4%)	1.000
**Weight (g)**	**Survivors**	**Nonsurvivors (30-day)**	**p-value**
	3115 (2820-3440)	2740 (2500-2990)	0.0257
**Age (days)**	**Survivors**	**Nonsurvivors (30-day)**	**p-value**
	3.0 (1.0-147.0)	3.0 (2.0-5.0)	0.1893

### Anatomy

Anatomical characteristics were described with regard to the size of the atrial septal defect, the anatomy of the mitral and aortic valves, and the size of the ascending aorta and patent ductus arteriosus. For patients with a single atrial septal defect (ASD), the median size of the defect was 3.55 (2.65-4.73) mm. For patients presenting multiple atrial septal defects, the total estimated ASD area was 10.8 (6.1-18.1) mm^2^. The mitral valve was normal in 1.3% (n=1) of the patients, stenotic in 53.7% (n=43), atretic in 43.7% (n=35), and one case had a single atrioventricular valve. The aortic valve was normal in 2.5% of patients (n=2), stenotic in 30% (n=24), and atretic in 67.5% (n=54). The ascending aorta size was 2.7 (2.0-4.3) mm and the size of the patent ductus arteriosus was 5.8 (5.00-6.5)mm. Anatomy variables from the survivor and the non-survivor patient groups were similar ( Table 2 ). We found no significant differences regarding the patients’ anatomy.

**Table 2:** Anatomical characteristics

ASD			
Area (mm^2^)	Survivors	Nonsurvivors (30-day)	p-value
Total estimated ASD area	10.95 (5.93-18.10)	8.7 (7.10-20.40)	0.7714
**Heart valves (LEFT)**			
**Mitral valve**	**Survivors**	**Nonsurvivors (30-day)**	**p-value**
normal	1/72 (1.4%)	0/7 (0.0%)	0.2187
stenosis	37/72 (51.4%)	6/7 (85.7%)	
atresia	34/72 (47.2%)	1/7 (14.3%)	
**Aortic valve**	**Survivors**	**Nonsurvivors (30-day)**	**p-value**
normal	2/73 (2.7%)	0/7 (0.0%)	0.2508
stenosis	20/73 (27.4%)	4/7 (57.1%)	
atresia	51/73 (69.9%)	3/7 (42.9%)	
**Subgroups**	**Survivors**	**Nonsurvivors (30-day)**	**p-value**
MS/AS	17/73 (23.3%)	4/7 (57.1%)	0.3267
MS/AA	19/73 (26.0%)	2/7 (28.6%)	
MA/AS	2/73 (2.7%)	0/7 (0.0%)	
MA/AA	32/73 (43.8%)	1/7 (14.3%)	
other	3/73 (4.1%)	0/7 (0.0%)	
**Aorta**			
**Ascending aorta**	**Survivors**	**Nonsurvivors (30-day)**	**p-value**
size (mm)	2.80 (2.00-4.30)	2.00 (2.00-6.20)	0.6612
patent ductus arteriosus	Survivors	Nonsurvivors (30-day)	p-value
size (mm)	5.75 (5.00-6.50)	6.70 (4.50-7.30)	0.5569

*NA: not applicable. ASD: atrial septal defect.*

### Operative data

The shunt diameter in the two patients who underwent the classic Norwood operation was 3.5 mm. In those who were submitted to the Norwood-Sano operation, a 4.0 mm (n=21; 27%) or a 5.0 mm graft (n=57; 73%) was selected. In 25 (32.4%) patients, shunt banding was employed. The median CPB, cross-clamp and circulatory arrest times were respectively 188 (170-214) min, 76 (70-80) min, and 48 (45-53) min. No significant difference was found between the surviving and non-surviving patient groups ( Table 3 ).

**Table 3:** Surgery

Shunt diameter (mm)	Survivors	Nonsurvivors (30-day)	p-value
3.5	1/41 (2.4%)	0/3 (0.0%)	0.9403
4.0	11/41 (26.8%)	1/3 (33.3%)	
5.0	29/41 (70.7%)	2/3 (66.7%)	
**Banding**	**Survivors**	**Nonsurvivors (30-day)**	**p-value**
patients undergoing shunt banding	11/32 (34.4%)	0/2 (0%)	0.3134
**Surgery times**	**Survivors**	**Nonsurvivors (30-day)**	**p-value**
CPB (min)	185 (170-210)	205 (180-240)	0.2202
Cross-clamp (min)	76 (70-80)	77 (59-82)	0.7057


*Obs.: There were missing data for the variables “shunt size” and “shunt banding” for 36 and 44 patients respectively. For this reason, we did not use these variables in uni or multivariate analysis. CPB: cardiopulmonary bypass.*

### Early and intermediate mortality, and ECMO support

Within the first 30 postoperative days, 7 patients (8.7%) died, resulting in an early survival rate of 91.3% ( Figure 1A ). Also, during these first 30 days, 14 patients (17.5%) required ECMO support. Among the survivors, only 13.7% received ECMO, compared to 57.1% of non-survivors (p=0.0039). The stratified survival curves for patients requiring or not requiring ECMO are illustrated in Figure 1B . The comparison of the survival curves indicates a worse outcome for those requiring circulatory support (Log-rank test, p=0.0020).

**Figure 1:** Early (up to 30 post-operative days) survival rates following the stage I Norwood procedure. A) Whole cohort (n=80). B) Comparison between ECMO (n=14) vs no ECMO (n=66) patients. ECMO: extracorporeal membrane oxygenation.

The intermediate survival rate was 81.3% once 8 additional patients died between the postoperative day 30 and the Glenn procedure ( Figure 2A ). ECMO was employed in 33,3% (n=3) of the 8 non-survivors, in contrast to 13,8% (n=9) of those who received the Glenn operation. Fig. 2B shows that the ECMO supported patients had a worse outcome as compared to those not requiring ECMO (Log-rank test, p=0.0088).

**Figure 2:** Intermediate survival rates (from 30 post-operative days through the Glenn procedure). A) Whole cohort (n=73). B) Comparison between ECMO (n=9) vs no ECMO (n=64) patients. ECMO: extracorporeal membrane oxygenation.

Irreversible neurological injury occurred in 4 children (28%) submitted to ECMO treatment. Dialysis was necessary in 85% (n=68) of the cases. All survivors recovered renal function. Post-operative aortic coarctation demanded reintervention in 6 (7.5%) patients (percutaneous stent placement, n=4; surgical enlargement simultaneous with the Glenn procedure, n= 2).

### Predictors of ECMO assistance, 30-day operative mortality, and intermediate mortality

Table 4 through 6 explore the potential predictors for ECMO assistance, early and intermediate mortality, respectively. Shunt size and bandage could not be analyzed due to missing data. By univariate analysis, CPB time was the only predictive factor for ECMO assistance, as no other variable reached the p< 0.250 threshold ( Table 4 ). For this reason, a multivariate analysis could not be carried out, and no variable could be confirmed as an independent ECMO predictor.

**Table 4:** ECMO assistance predictors according to univariate and multivariate logistic regression

	univariate		multivariate	

	OR (95%CI)	p-value	OR (95%CI)	p-value
**Demographics**				
Sex (for male)	1.524 (0.432-5.383)	0.513	-	-
Weight (per g)	1.000 (0.998-1.001)	0.515	-	-
Age (per day)	0.860 (0.606-1.220)	0.397	-	-
**Anatomy**				
Total estimated ASD area (per mm^2^)	0.982 (0.944-1.021)	0.354	-	-
Mitral valve (atresia vs. stenosis)	0.905 (0.282-2.909)	0.867	-	-
Aortic valve (atresia vs. stenosis)	1.791 (0.451-7.112)	0.408	-	-
Asc Ao size (per mm)	0.907 (0.635-1.295)	0.590	-	-
**Surgery**				
CPB (per min)	1.006 (0.996-1.016)	0.248	1.006 (0.996-1.016)	0.248
Cross-clamp (per min)	0.997 (0.944-1.054)	0.922	-	-

*ASD: atrial septal defect; CPB: cardiopulmonary bypass.*

In regard to 30-day postoperative mortality ( Table 5 ), body weight, mitral and aortic valve anatomy, CPB time, and ECMO assistance were considered predictive factors by the univariate analysis. However, by multivariate analysis, the mitral valve anatomy and the CPB time did not hold as independent predictors, in contrast to body weight, the aortic valve anatomy and the need for ECMO support, which were confirmed as independent risk factors. The greater the weight, the lower the risk (OR 0.997 per gram; 95% CI 0.995- 1.000; p=0.033). Aortic valve atresia was a protective factor as compared to stenosis (OR 0.090; 95% CI 0.009-0.857; p=0.036), and the need for post-operative ECMO was an important independent risk factor for mortality (OR 20.975; 95% CI 2.116-207.886; p=0.009). Shunt size and Sano tube banding could not be analyzed by uni/multivariate logistic regression due to missing data.

**Table 5:** 30-day mortality predictors according to univariate and multivariate logistic regression

	univariate		multivariate	

	OR (95%CI)	p-value	OR (95%CI)	p-value
**Demographics**				
Sex (for males)	1.467 (0.266-8.091)	0.660	-	-
Weight (per g)	0.998 (0.996-1.000)	0.056	0.997 (0.995-1.000)	0.033
Age (per day)	0.734 (0.382-1.413)	0.355	-	-
**Anatomy**				
total estimated ASD area (per mm^2^)	1.010 (0.983-1.037)	0.465	-	-
mitral valve (atresia vs. stenosis)	0.181 (0.021-1.585)	0.123	0.491 (0.024-10.089)	0.645
aortic valve (atresia vs. stenosis)	0.294 (0.060-1.433)	0.130	0.090 (0.009-0.857)	0.036
Asc Ao size (per mm)	0.981 (0.620-1.552)	0.935	-	-
**Surgery**				
CPB (per min)	1.010 (0.999-1.021)	0.089	1.018 (0.992-1.044)	0.173
Cross-clamp (per min)	0.952 (0.874-1.037)	0.260	-	-
ECMO	8.400 (1.631-43.256)	0.011	20.975 (2.116-207.886)	0.009

*ASD: atrial septal defect; CPB: cardiopulmonary bypass; ECMO: extracorporeal membrane oxygenation.*

By univariate analysis, mitral and aortic valve anatomy, CPB time, and ECMO support were predictive factors for intermediate mortality ( Table 6 ). In the multivariate analysis, however, the mitral valve anatomy came up as the only predictor for mortality. Valvar stenosis led to a worse prognosis as compared to valve atresia (OR 0.242; 95% CCI 0.062-0.942; p=0.041).

**Table 6:** Intermediate mortality predictors according to univariate and multivariate logistic regression

	univariate		multivariate	

	OR (95%CI)	p-value	OR (95%CI)	p-value
**Demographics**				
Sex (for males)	1.719 (0.493-5.991)	0.395	-	-
Weight (per g)	1.000 (0.998-1.001)	0.511	-	-
Age (per day)	1.008 (0.986-1.031)	0.457	-	-
**Anatomy**				
Total estimated ASD area (per mm^2^)	1.010 (0.989-1.031)	0.354	-	-
Mitral valve (atresia vs. stenosis)	0.242 (0.062-0.942)	0.041	0.242 (0.062-0.942)	0.041
Aortic valve (atresia vs. stenosis)	0.422 (0.133-1.343)	0.144	0.357 (0.059-2.174)	0.264
Asc Ao size (per mm)	1.003 (0.723-1.392)	0.984	-	-
**Surgery**				
CPB (per min)	1.008 (0.998-1.018)	0.131	1.017 (0.998-1.037)	0.080
Cross-clamp (per min)	0.992 (0.936-1.051)	0.785	-	-
ECMO	3.111 (0.862-11.231)	0.083	3.011 (0.623-14.542)	0.170

*ASD: atrial septal defect; CPB: cardiopulmonary bypass; ECMO: extracorporeal membrane oxygenation.*

## Discussion

Since the establishment of the classic Norwood or the Norwood-Sano procedures as the standard surgical management for the treatment of patients with HLHS, there has been a progressive worldwide improvement in the survival rate. The present study included 80 consecutive patients operated on from 2016 who were extracted from our group series of more than 500 patients to represent current early outcomes in the era of ECMO support. Our 8.7% early mortality rate for patients undergoing the Norwood/Norwood-Sano procedures is among the lowest reported.^10 , 11^ Others have reported a 30-day postoperative mortality of 15.2%.^12^ Interim mortality (from after hospital discharge following the Norwood procedure through the Glenn operation) varies from 5-28%.^13^ According to the Society of Thoracic Surgeons’ (STS’s) Congenital Heart Surgery Database,^20^ overall mortality is 22%, while the mortality for patients with any complication (27%) is much higher (p<0.0001) as compared to patients who did not suffer a complication (7%).

In the present study, low body weight was found as an independent predictor for early mortality following the stage I Norwood operation, in accordance with what has been described by several previous studies.^12 , 16 , 21 , 22^

The aortic and mitral valve anatomy have also been recognized as predictors of early mortality in previous studies.^23^ The presence of aortic and/or mitral atresia is usually associated with higher mortality rates, particularly when mitral stenosis is accompanied by aortic atresia,^23 , 24 , 29^ or is associated with a restrictive ASD.^25^ In the present investigation, both the aortic valve anatomy and the need for ECMO assistance showed up as independent risk factors for early mortality. Curiously, in our study, aortic valve atresia, as compared to aortic valve stenosis, was a protective factor against mortality, and the same was true concerning the mitral valve anatomy.

Furthermore, we detected the need for ECMO support as an important independent risk factor for early mortality. Indeed, ECMO supported patients had a >20-times mortality risk than patients who did not need mechanical circulatory support. Unfortunately, we could not isolate any independent predictive factor for ECMO assistance, although previous studies reported birth weight <2.5 kg and longer CPB time as independently associated with the need for ECMO after the Norwood operation.^30^

In the present study, mitral valve atresia and prolonged CPB times turned up as predictors for early mortality by univariate analysis, but they were not confirmed as independent predictors for early mortality by multivariate analysis. When intermediate mortality was examined, only the mitral valve anatomy came out as an independent risk factor, mitral valve stenosis correlating with a worse prognosis as compared to valve atresia. Prolonged CPB times have not been reported as a mortality predictor in the Norwood procedure,^14 , 16 , 18^ although some studies have reported borderline p- values.

Two other important mortality predictors of the stage I Norwood operation are the center and surgeon surgical volumes. Both these variables have been significantly associated with outcomes following the Norwood procedure according to the STS’s Congenital Heart Surgery Database.^31^ The STS reported that centers operating on more than 20 cases per year and surgeons operating on more than 10 cases per year presented lower mortality rates. The present study confirms the same holds true outside North America, as the low mortality rates herein reported derived from both high center and surgeon caseloads.

### Limitations

We recognize that our study has some limitations. The retrospective analyses made it impossible to avoid data missing, as clinical information recording was being transitioned from manual to digital over the study period. Outcomes might have been negatively affected once ECMO indication was more conservative and sometimes delayed early in the series, when an in-house ECMO team was not yet available. Accordingly, the interstice between ECMO indication and ECMO support initiation was much reduced later in the series.

## Conclusion

The present study reports a large Brazilian cohort of patients with HLHS undergoing the Norwood procedure in the recent era. We had a 30-day survival rate of 91.3%, which is comparable to the highest survival rates reported worldwide and an intermediate survival rate of 81.3%. Low body weight, aortic stenosis (as compared to aortic atresia), and the need for ECMO support were independent predictors of 30-day mortality, whereas mitral and aortic valve anatomy, CPB time, and ECMO support were predictive factors for intermediate mortality. No independent risk factor for ECMO support could be evidenced. Future studies targeting the interstage mortality, as well as the mortality of other procedures involved in the palliative reconstruction of HLHS, may provide additional evidence for the long-term survival rate and add other potential predictive factors for mortality.
